# Chiral Molecular Coating of a LiNiCoMnO_2_ Cathode for High-Rate Capability Lithium-Ion Batteries

**DOI:** 10.1021/acs.jpclett.4c00171

**Published:** 2024-03-01

**Authors:** Nir Yuran, Bagavathi Muniyandi, Arka Saha, Shira Yochelis, Daniel Sharon, Yossi Paltiel, Malachi Noked

**Affiliations:** † Department of Applied Physics, Center for Nanoscience and Nanotechnology, Hebrew University of Jerusalem, Jerusalem 91904, Israel; ‡ Department of Chemistry, Bar Ilan Institute for Nanotechnology and Advanced Materials, Bar Ilan University, Ramat Gan 5290002, Israel

## Abstract

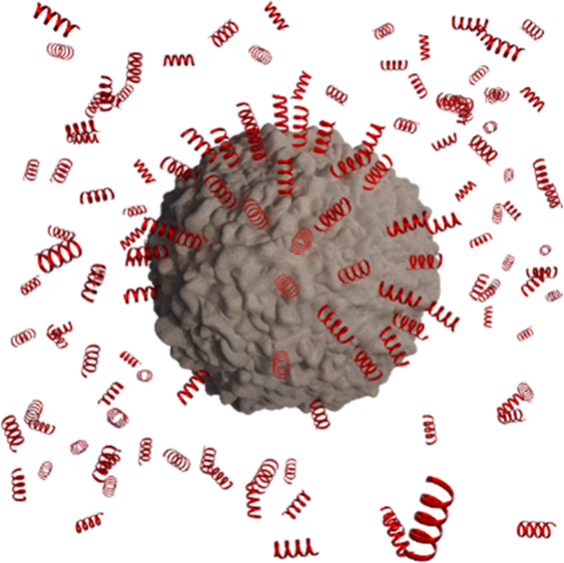

The growing demand for energy has increased the need for battery storage, with lithium-ion batteries being widely used. Among those, nickel-rich layered lithium transition metal oxides [LiNi_1–*x–y*_Co_*x*_Mn_*y*_O_2_ NCM (1 – *x* – *y* > 0.5)] are some of the promising cathode materials due to their high specific capacities and working voltages. In this study, we demonstrate that a thin, simple coating of polyalanine chiral molecules improves the performance of Ni-rich cathodes. The chiral organic coating of the active material enhances the discharge capacity and rate capability. Specifically, NCM811 and NCM622 electrodes coated with chiral molecules exhibit lower voltage hysteresis and better rate performance, with a capacity improvement of >10% at a 4 C discharge rate and an average improvement of 6%. We relate these results to the chirally induced spin selectivity effect that enables us to reduce the resistance of the electrode interface and to reduce dramatically the overpotential needed for the chemical process by aligning the electron spins.

Lithium-ion batteries (LIBs) are the most common type of electrochemical energy storage used in a variety of industries, including electric vehicles, phones, portable electronics, and stationary grid power stations.^1,2^ They are known for their high energy density, reliability, and efficiency.^3−6^ Nickel-rich layered lithium transition metal oxides are promising cathode materials for next-generation LIBs used in automotive applications because of their high specific capacities (180–220 mAh/g), high working voltages (typically 3.6–3.7 V), good rate capabilities, and relatively low cost.^7−9^ However, these materials can suffer from structural and interfacial instability during repeated charging and discharging, leading to deterioration of performance and safety concerns. One issue is that the highly reactive materials can accelerate the decomposition of electrolytes, resulting in rapid capacity fading and overall poor battery performance.^7,10^ The internal resistance and overpotential also play crucial roles in battery performance. A low electronic resistance leads to a higher power density and a lower risk of overheating, while a low overpotential results in a higher energy density.^11^ The capacity fading and relative insufficient rate capability would become severe effects due to the high Ni content in NCM cathode materials during operation at a higher cutoff voltage (>4.2 V vs Li^+^/Li).^12^ The capacity fading can be attributed to structural degradation.^13,14^ Undesired transition metal dissolution from the cathode could destroy the structural stability of the cathode active materials and alter the composition of the solid electrolyte interphase (SEI) at the anode side.^15^

Therefore, a great deal of effort has been dedicated to improving the safety and performance of batteries. Promising approaches utilize surface modification of the cathode material by a coating process.^16,17^ This approach aims to address the capacity fading of Ni-rich cathode materials during long charge–discharge cycling. The protective surface modifications include both cathode and anode surface coating using a core–shell structural design by wet chemical methods,^18^ physical vapor deposition,^19^ chemical vapor deposition,^20^ and atomic layer deposition.^21,22^ The surface treatment can protect the interface between the NCM cathode material and electrolyte, suppressing transition metal dissolution from the cathode and reducing side reactions for improving the electrochemical performance in terms of rate capability, retention of specific capacity, and long-term cycling.^23,24^ Moreover, the coating may reduce the number of microcracks and the rate of oxygen release that would take hold of these changes after long charge–discharge cycles, which introduces safety defects.^25^

This approach was widely investigated in lithium-ion batteries with metal oxides such as Al_2_O_3_, SiO_2_, TiO_2_, ZnO, and ZrO_2_, phosphates (AlPO_4_ and Li_3_PO_4_), fluorides (AlF_3_),^26,27^ polymeric materials,^28^ and metal–organic frameworks (MOFs),^29,30^ which are protected on cathode materials because of their resisting abilities to avoid the direct electrode–electrolyte contact and corrosion of HF on cathode materials during long charge–discharge cycles. However, most of these inorganic coating materials have impecunious electrical conductivities and parasitic byproduct formation with lithium and cathode materials, which show a conflicting effect on the electrochemical performance. In addition, most of these coating materials are Li^+^-ion insulators, which inhibit the diffusion of Li^+^ ions in the cathode material and, therefore, prevent full capacity operation.

Our unique and simple approach involves a molecular nanometric chiral coating that is known to enable pure spin current because of the chirally induced spin selectivity (CISS) effect.^31−34^ Chirality is a property of objects that cannot be superimposed onto their mirror image, much like the case for left and right hands. Chiral molecules are essential in chemistry and biology as they can have different properties and reactivities compared to those of their mirror image.^35,36^ The CISS effect is a phenomenon in which the spin state of electrons passing through a chiral molecule is selectively affected by the molecule’s handedness.^31,33,37,38^ In other words, the spin of electrons passing through a left-handed molecule differs from the spin state of electrons passing through a right-handed molecule.^31,33^ Thus, charge displacement and transmission in chiral molecules generate a spin-polarized electron distribution.^39,40^ A spin-polarized electron cannot backscatter in the chiral potential, and therefore, the resistance is reduced.^41^

The electron spin is also critical in chemical reactions in which most bonds are in a singlet state.^42,43^ However, the oxygen molecule is special with a triplet state at the ground energy level.^42^ Therefore, standard oxidation processes are spin forbidden and have a large overpotential. In these cases, the CISS effect can be utilized to align multiple electron spins enabling the enhancement of the efficiency of these processes.^44^ Similarly, spin alignment can be used to increase the frequency of obtaining a spin selective current in electrolyzers and fuel cells, improving their efficiency.^45^ Indeed, it was demonstrated that using chiral molecules as intermediaries in water splitting can increase the efficiency by decreasing the overpotential by 50%.^44,45^ In the work presented here, the electrochemical properties of electrodes coated with chiral l-α-helix polyalanine 36 and 16 (AHPA), [H]-C(AAAAK)_7/3_-[OH] (C, A, and K represent cysteine, alanine, and lysine, respectively), and achiral 12-mercaptododecanoic acid (MDA) (molecular structures described in Figure S1), both purchased from Sigma-Aldrich, Ltd., Israel, and pristine uncoated cathode materials were compared. The chiral molecules coated the active material, and the results indicate an increase in specific capacitance at both low and high charge and discharge rates using the chiral coating. Specifically, the AHPA chirally coated NCM811 and NCM622 cathode material enhances the efficiency by 6–14%, decreasing the overpotential by 0.1 V in the reduction process and reducing the energy loss and heating obstacles.

## Characterization of Materials

The crystal structures of samples were determined using X-ray diffraction (XRD, D8 advance, Bruker). The chemical compositions of the coating layers with cathode materials were analyzed by X-ray photoelectron spectroscopy (XPS) (Thermo Scientific K-Alpha) under Al Kα radiation. The X-ray photoelectron spectroscopy (XPS) measurements were performed in UHV (2.5 × 10^–10^ Torr base pressure) using the 5600 Multi-Technique System. The samples were irradiated with an Al Kα monochromatic source (1486.6 eV), and the departing electrons were analyzed by a spherical capacitor analyzer using a slit aperture of 0.8 mm. The morphology of the samples was analyzed using a high-resolution scanning electron microscopy (HRSEM) Magellan 400Lis instrument from FEI. The morphologies and microstructures of the samples were detected by field emission scanning electron microscopy (Nova Nano SEM 450, FEI) and high-resolution transmission electron microscopy (HRTEM) (Tecnai G2 F20, FEI).

**Figure 1:**
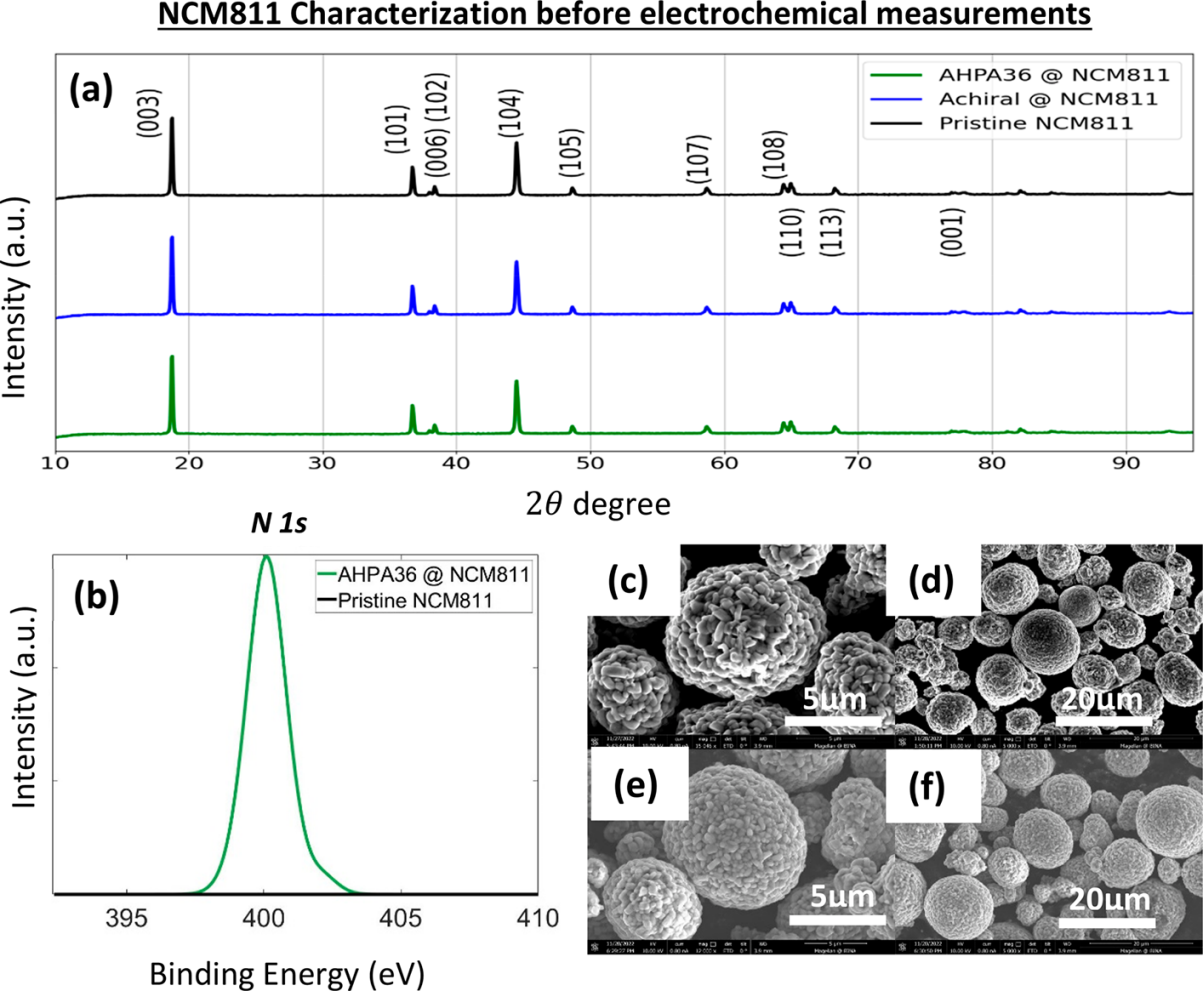
XRD peaks of treated and pristine NCM powder, XPS spectrum of treated and untreated NCM particles, and high-resolution SEM (HRSEM) images. (a) XRD patterns of the NCM powder samples in the 2θ range of 10–90°. (b) N 1s XPS spectra of AHPA36@NCM811 and pristine NCM811. Low- and high-magnification images of (c and d) AHPA36@NCM811 and (e and f) pristine NCM811 powder samples.

Figure 1b presents the XPS spectrum of AHPA36@NCM811 and pristine NCM811. The results imply that the organic material was adsorbed on the NCM particles. Figure 1b presents the typical N 1s XPS spectrum, in which a peak at 400.1 eV associated with pyrrolic N is observed and can be related to the amino groups in the chiral polypeptides.

Figure 1a shows that the XRD peaks of the NCM powder samples are in the 2θ range of 10–90°. All diffraction peaks are well indexed with the respective crystal planes of the hexagonal crystal structure in space group *R*3̅*m*. The measurements show that the adsorption of molecules does not seem to change the NCM properties. All of the diffraction peaks are indexed to the hexagonal α-NaFeO_2_ phase, and there is no noticeable change in the XRD patterns of coated samples and pristine NCM, proving the similar crystal structure of cathode materials before and after surface modification. The characteristic diffraction peak position of treated and pristine NCM samples is also consistent with the literature, which means that the NCM samples with a layered structure can be successfully retained after the chiral protective coating on pristine NCM811.

The results also indicate an absence of impurities in the NCM powder samples, suggesting a low content of molecules on the surface of NCM particles passivating the surface.^46^ In general, the intensity ratio of the (003) and (104) diffraction peaks can be used to determine the order of cations in NCM materials, and the ratio value is inversely proportional to the cationic order. The layered structure with a lower degree of cation mixing is more stable when the ratio is >1.2. The calculated *I*(003)/*I*(104) ratios for coated AHPA36@NCM811, coated achiral@NCM811, and pristine NCM811 are 1.35, 1.35, and 1.58, respectively.^25^ In addition, the (006)/(102) and (108)/(110) double splitting peaks represent the degrees of order of the material crystal structure, which has not been affected by the chirally protected cathode materials. Both results confirm that the materials have a layered structure.

The HRSEM images of all three treated samples, AHPA36@NCM811, achiral@NCM811, and pristine NCM811, are presented in Figure 1c–f and in the Supporting Information. These images show that the chiral and achiral coating on NCM811 particles has not affected or degraded the cathode materials via adsorption and that the particles’ spherical structures were preserved. The elemental maps of the individual particles demonstrate that the coating covers the NCM particle surface (Figures S11–S13). The uniform thin layer of AHPA36 coating on NCM811 is confirmed by the transmission electron microscopy images (Figure S10). It is important to note that in Figure S11 the nitrogen indicates adsorption of the amine group on the active material.

**Figure 2:**
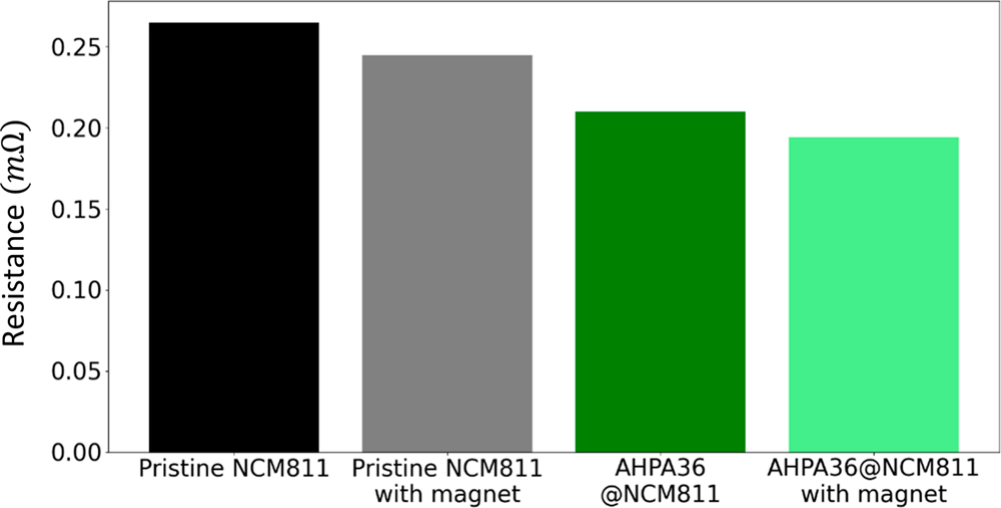
Resistance at 1 V of the coated and pristine NCM811 material layer adsorbed to a magnetic electrode. The resistance in 1 V of the coated active material is reduced by 25%, while aligning the magnetic field with the current reduces the resistance by an additional 10%. The magnetic substrate is Ni with in-plane easy axis. The error range is ±1.5 μΩ.

It is important to appreciate that the coated active material with chiral polyalanine reduces resistance, as expected from the CISS effect, and acts as a spin filter (Figure 2). Figure 2 presents the resistance of the NMC811 active material with and without a chiral coating when the material is adsorbed to a magnetic Ni electrode. The resistance of the coated material is reduced by 25% due to the CISS effect. Out-of-plane magnetization parallel to the current flow reduces further the resistance by an additional 10%, which is related to spin filtering. The *I–V* curves are presented in Figure S6.

**Figure 3:**
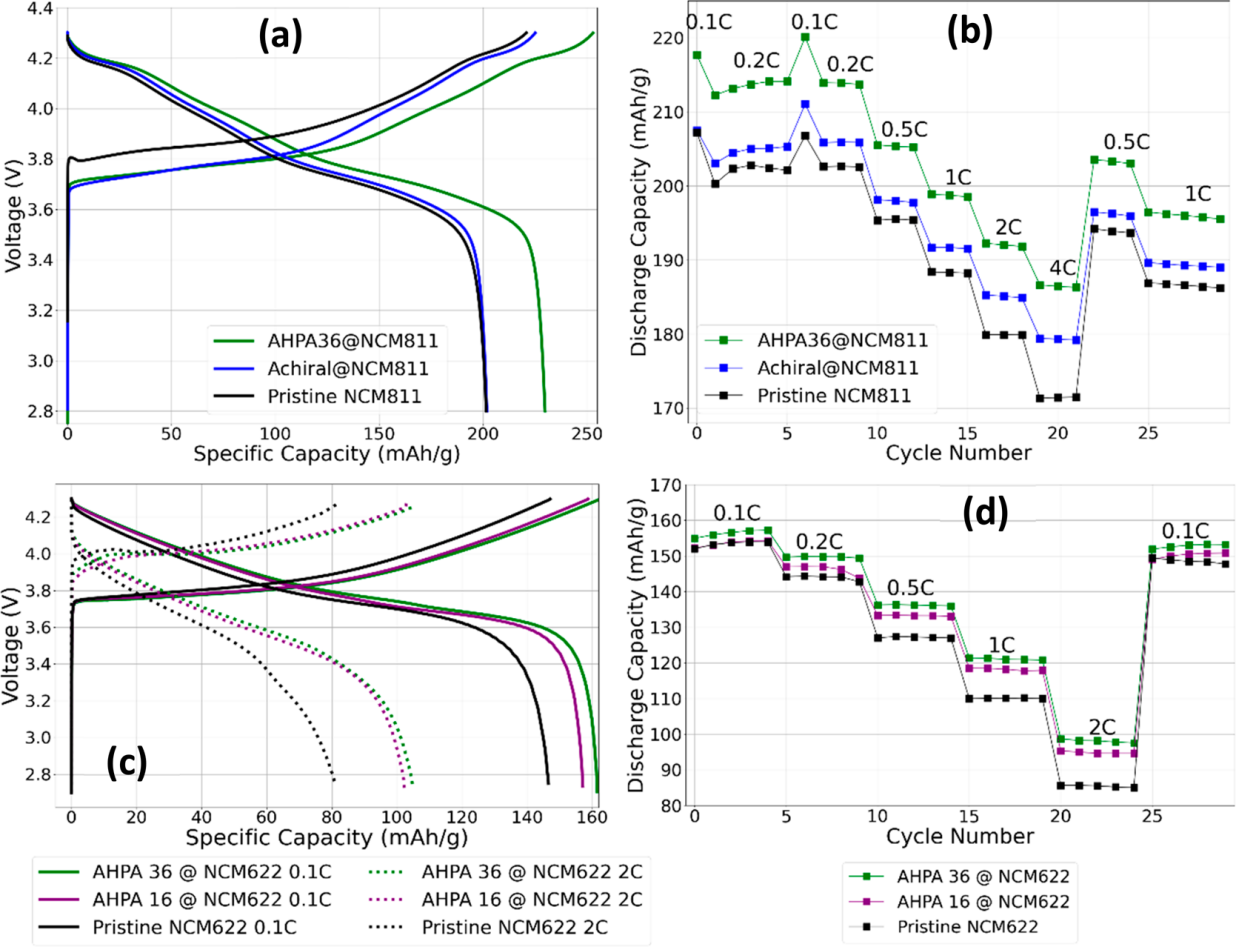
Electrochemical measurements of NCM811. Measurements of AHPA36@NCM811, achiral@NCM811, and pristine NCM811/Li half-cells for (a) charge–discharge voltage profiles and (b) discharge capacity rates profiles. Measurements of the polyalanine chiral coating of different lengths of AHPA36 and AHPA16 and pristine NCM622/Li half-cells for (c) charge–discharge voltage profiles and (d) discharge capacity rates profiles.

The voltage profiles of uncoated NCM811 (pristine) and coated NCM811 electrodes (vs a Li counterelectrode) are presented in Figure 3a. Both uncoated and coated NCM811 samples exhibit two voltage plateaus at ∼3.7 and ∼4.2 V. The overlapped voltage profiles of the different samples in Figure 3a show two main observations. First, a lower average overpotential was obtained with chirally coated NCM811 with respect to the achirally coated or uncoated NCM811. Second, cells with chirally coated NCM present a specific discharge capacity that is higher than that of the achirally coated or uncoated NCM.

The performance of the samples was evaluated in the voltage range of 2.8–4.3 V using coin-type CR2032 lithium half-cells at rates ranging from 0.1 C (discharge at 10 h) to 4 C (discharge at 15 min). The initial charge–discharge formation voltage profile of all samples at a rate of 0.1 C is shown in Figure 3b. The specific discharge capacities for the AHPA36@NCM811, achiral@NCM811, and pristine NCM811 cathodes are 217.7 ± 1, 207, and 206 mAh/g, respectively. As one can see, the rate performance is improved by the surface coating. At low C rates (0.1 and 0.2 C), the AHPA36 sample NCM811 delivered specific discharge capacities (5–10 mAh/g) that were larger than those of the other samples.

A more significant enhancement of discharge capacity is observed at higher rates of 1, 2, and 4 C. Overall, the electrochemical performance of the protected NCM cathode materials, AHPA36@NCM811 and achiral@NCM811, shows rate capabilities better than that of pristine NCM811. Compared to that of the uncoated sample, the specific discharge capacities of the AHPA36@NCM811 sample show an average increase of 8.7 ± 0.3% in the specific discharge capacities at 4 C and 6 ± 0.2% on average. Achiral@NCM811 shows a smaller increase in the specific discharge capacities of NCM811, which in general improves the kinetics of the Li intercalation/deintercalation processes as both chiral and achiral coatings improve performance. This may imply that the organic coatings can reduce electrolyte solution breakdown on the NCM surface, forming a thick passivation layer that slows Li intercalation/deintercalation kinetics. However, the cells with chiral coatings outperform those with achiral coatings, indicating that in addition to physical protection, the chiral coating enhances the discharge/charge processes. An additional sample has been prepared, for which the excess chiral molecules (AHPA36) were filtered using 3 μm filter paper, leading to a smaller amount of AHPA36 in the active material slurry (see Figure S4d–f for AHPA36@NCM811 B). Two samples have been prepared for each coating and the pristine form (see Figure S4). These measurements show that reduction of the polyalanine coating density reduces the improvement in capacitance, linking the coating itself with an improvement in efficiency.

The charge–discharge voltage profiles of all three cathodes were stable with respect to the specific capacity of different cycles, which is shown in Figure S2. The thickness of the coating strongly influences the electrochemical performance of the cathode materials. Thin coating layers (1–3 nm) are not sufficient to protect NCM811 completely, and its ability to hinder the side reactions at the interface is poor. A thicker and more stable coating may help.

To validate the impact of the chiral coating on the augmentation of battery capacity, we conducted experiments using another active material, namely, NCM622, along with varying lengths of chiral molecules, specifically α-helix polyalanine 16 (AHPA 16). The active material composition of the test samples is as follows: AHPA36@NCM622 weighing 11 mg, AHPA16@NCM622 weighing 10 mg, and pristine NCM622 weighing 9 mg. The voltage profiles were measured at a discharge rate of 0.1 C. Three samples were measured for each coating and the pristine sample, while each measurement was performed at least twice at the lab in Israel or at the lab in the United States (Figure S5).

Panels c and d of Figure 3 present a similar enhancement following the same procedure that was used for AHPA36@NCM811. Figure 3c shows a reduction of ∼0.5 V in the overpotential at 2 C for AHPA36@NCM622 compared to that of pristine NCM622. Figure 3d shows an overall increase in specific capacity and a greater enhancement at high discharge rates, specifically 2 C. For AHPA36@NCM622, we observed an average capacity increase of 6 ± 0.2%, with a 14 ± 0.3% improvement at 2 C. Similarly, AHPA16@NCM622 exhibited an average capacity increase of 3.7 ± 0.2%, with a 9% enhancement at 2 C. It is important to note that longer polypeptides have better spin polarization,^47^ matching the better performance we achieved with AHPA 36.

**Figure 4:**
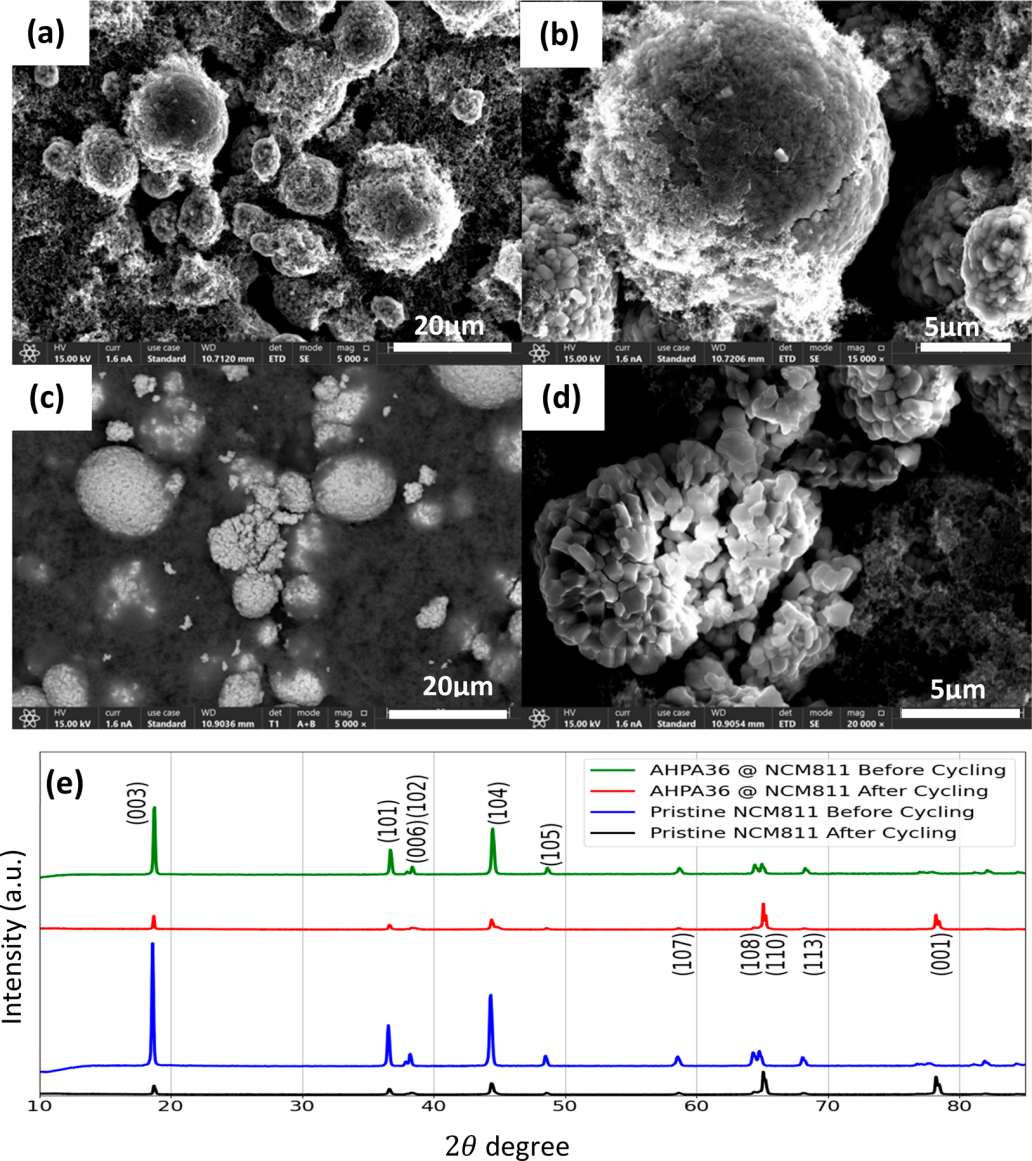
Post-mortem analysis. SEM of cycled electrodes at 1 C of (a and b) AHPA36@NCM811 and (c and d) pristine NCM811. (e) Comparative XRD patterns of pre- and postcycling electrodes of AHPA36@NCM811 and pristine NCM811 samples.

*Characterization of AHPA36@NCM811 and Pristine NCM811 after Electrochemical Cycling*. Lastly, the morphology and composition of the cathode materials were further investigated using HRSEM and EDX spectroscopy after cycling the electrodes shown in Figure 4a–d. The treated AHPA36@NCM811 electrode does not have the crack and structural degradation during the electrochemical charge–discharge cycling of the core NCM811 particles. In the case of pristine samples, a clear degradation of primary particles from the secondary macro-sized particles is observed with the formation and erosion of a few cracks. The EDX spectral analysis data of AHPA36@NCM811 and pristine NCM811 cathodes confirmed the retention of the NCM811 composition after continuous charge–discharge cycling.

From the post-mortem HRSEM and EDX analysis, the chiral coating may provide protection to the NCM cathode materials, reducing the extent of direct contact with electrolytes during the long charge–discharge cycling process and reducing bulk process. The X-ray diffraction pattern shown in Figure 4e of the protectively chirally coated AHPA36@NCM811 and pristine NCM811 electrodes shows that no structural and phase changes are observed in any of the samples after continuous cycling. More morphological and structural effects of coated and uncoated samples compared before and after cycling are presented in Figures S11 and S12.

The results presented here aligned with those of previous studies showing improvement in oxygen reduction following the coating of the anode with a chiral material.^44,45,48^ This improvement in efficiency was ascribed to the CISS effect.^33,49,50^ Due to the chiral coating, spin alignment of the injected current is achieved (Figure 2). The spin current reduces the junction resistance (Figure 2) due to angular momentum conservation, while also minimizing the overpotential needed to generate the triplet ground O_2_ state. The surface is becoming more active, reducing bulk material trapping and overpotential heating. The coating also prevents the generation of side products that shorten the lifetime of the electrodes. In Li-ion batteries, similar effects are also expected to occur.

Following adsorption, there is no apparent change in the NCM structure. AHPA acts as an intermediate material of the SEI. The length of the AHPA is 5.4 nm on top of micrometer-size particles, which is negligible when changes in surface area are calculated. Therefore, the chiral coating should reduce the electrode surface resistance while adding a small amount of material. Even more importantly, the spin alignment could decrease the overpotential in multielectron processes. We believe that when we cover the surface with chiral molecules, trapping of oxide in the bulk material is prevented as the surface becomes much more active as spins are aligned, and the resistance is reduced.

It is important to mention that some of the improved behavior of the coated NCM811 (NCM622) electrodes can be associated with the lower irreversibility (electrolyte solution degradation) of the initial cycle and improved kinetics of the intercalation and deintercalation processes due to the coating, unrelated to spin, as shown by the small improvement measured with achiral coatings.

**Figure 5:**
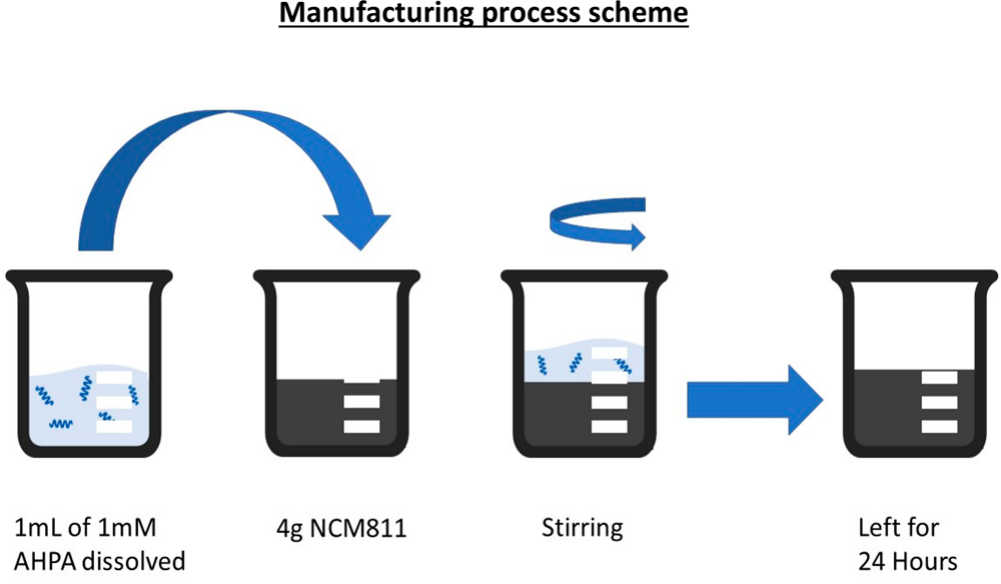
Coating flow process. The flow process includes four stages: UV treatment of the active material, coating of NCM811 (NCM622) powder with polyalanine, stirring of the material, and drying for 24 h. The process is simple and can be used with different active materials.

*Coating of the Active Material*. To study the effect of the CISS on Li-ion batteries, we adsorbed chiral AHPA molecules on the active material of the battery. The flow processes for coating cathode materials with chiral molecules are illustrated in Figure 5. Cathode powders of commercial LiNi_0.8_Mn_0.1_Co_0.1_O_2_ (NCM 811) purchased from TARGRAY-USA are subjected to ultraviolet (UV) treatment before being inserted into a 1 mM ethanol solution of l-α-helix polyalanine 36 (3008.71 g/mol). A total amount of 3 mg of APHA was used to coat 4 g of NCM811 (NCM622) powder. The reactor is gently shaken to homogenize the suspension during the coating process. After 24 h, the sample is dried under a nitrogen environment and coated NCM811 (NCM622) powder is obtained (AHPA36@NCM811). A control sample and a sample with AHPA16 were prepared in the same manner as the AHPA36@NCM811 sample but with achiral molecules [12-mercaptododecanoic acid (232.38 g/mol, Sigma-Aldrich) and AHPA16 (1348.78 g/mol) (achiral@NCM and AHPA16@NCM, respectively)]. The proposed procedure is extremely simple and can be easily scaled up for battery production.

*Cell Assembly and Electrochemical Measurements*. Electrochemical tests were carried out using the 2032 coin-type test cells. The cathode composed of a polyvinylidene fluoride (PVDF) binder (10 wt %), acetylene carbon black (10 wt %), and chirally coated NCM811 active material (80 wt %) was dispersed into *N*-methylpyrrolidone (NMP) to make a homogeneous slurry. The slurry was cast onto an aluminum foil, dried on a hot plate, and then dried at 110 °C under vacuum overnight to evaporate the NMP solvent. The dried cathode was cut into a circle with a diameter of 12 mm as the cathode electrode of the LIB coin cells, and the mass loading of active materials was ≈2.34 mg. Lithium foil (200 μm thick) and a Celgard PP2500 polypropylene membrane served as the anode and separator, respectively. A commercial electrolyte solution (LP-57) comprising 1 M LiPF_6_ in a 3:7 (v/v) ethylene carbonate/ethyl methyl carbonate mixture was used in this study as the electrolyte. The coin cells (CR-2032) were assembled in a glovebox filled with argon gas, and the moisture and oxygen contents were ≤0.1 ppm. The cycle and rate performance were galvanostatically determined within the voltage window of 2.8–4.3 V (vs Li/Li^+^) using a Neware battery tester at room temperature (30 °C). Cyclic voltammetry (CV) with a potential scan rate of 0.1 mV s^–1^ in the range of 2.8–4.3 V and electrochemical impedance spectroscopy (EIS) within a frequency range of 0.01–10^5^ Hz at a perturbation amplitude voltage of 5 mV were carried out on a Biologic (VSP) system.

In summary, our research has demonstrated that using a chiral coating technique to modify the surface of NCM811 and NCM622 materials can significantly improve the electrochemical performance of lithium-ion cells. The NCM811 (NCM622) electrodes coated with chiral molecules exhibited lower voltage hysteresis and better rate performance, with a capacity improvement of 10% at a 4 C (14% at 2 C) discharge rate and an average improvement of 6% in rate capability measurements. In comparison, achiral samples showed a capacity improvement of only 4.6% at 4 C and an average improvement of 2%. Coating with a longer chiral molecule improves the performance the most. These results demonstrate that the CISS effect plays a vital role in the charge–discharge process.

## Supporting Information Available

Figures S1–S13 and an additional reference (PDF)

## References

[ref1] Duan J.; Tang X.; Dai H.; Yang Y.; Wu W.; Wei X.; Huang Y. Building Safe Lithium-Ion Batteries for Electric Vehicles: A Review. Electrochemical Energy Reviews 2020, 3 (1), 1–42. 10.1007/s41918-019-00060-4.

[ref2] Goodenough J. B. C. Energy Storage Materials; Elsevier, 2015; Vol. 1, pp 158–161. 10.1016/j.ensm.2015.07.001

[ref3] Schipper F.; Erickson E. M.; Erk C.; Shin J.-Y.; Chesneau F. F.; Aurbach D. Review—Recent Advances and Remaining Challenges for Lithium Ion Battery Cathodes. J. Electrochem. Soc. 2017, 164 (1), A6220–A6228. 10.1149/2.0351701jes.

[ref4] Sun Y. K.; Myung S. T.; Park B. C.; Prakash J.; Belharouak I.; Amine K. High-Energy Cathode Material for Long-Life and Safe Lithium Batteries. Nat. Mater. 2009, 8 (4), 320–324. 10.1038/nmat2418. 19305398

[ref5] Sun Y.; Liu N.; Cui Y. Promises and Challenges of Nanomaterials for Lithium-Based Rechargeable Batteries. Nat. Energy 2016, 1, 16071 10.1038/nenergy.2016.71.

[ref6] Lim B. B.; Yoon S. J.; Park K. J.; Yoon C. S.; Kim S. J.; Lee J. J.; Sun Y. K. Advanced Concentration Gradient Cathode Material with Two-Slope for High-Energy and Safe Lithium Batteries. Adv. Funct Mater. 2015, 25 (29), 4673–4680. 10.1002/adfm.201501430.

[ref7] Manthiram A.; Song B.; Li W. A Perspective on Nickel-Rich Layered Oxide Cathodes for Lithium-Ion Batteries. Energy Storage Materials 2017, 6, 125–139. 10.1016/j.ensm.2016.10.007.

[ref8] Manthiram A.; Knight J. C.; Myung S. T.; Oh S. M.; Sun Y. K. Nickel-Rich and Lithium-Rich Layered Oxide Cathodes: Progress and Perspectives. Adv. Energy Mater. 2016, 6 (1), 1501010 10.1002/aenm.201501010.

[ref9] Liu W.; Oh P.; Liu X.; Lee M.-J.; Cho W.; Chae S.; Kim Y.; Cho J. Nickel-Rich Layered Lithium Transition-Metal Oxide for High-Energy Lithium-Ion Batteries. Angew. Chem. 2015, 127 (15), 4518–4536. 10.1002/ange.201409262. 25801735

[ref10] Xia Y.; Zheng J.; Wang C.; Gu M. Designing Principle for Ni-Rich Cathode Materials with High Energy Density for Practical Applications. Nano Energy 2018, 49, 434–452. 10.1016/j.nanoen.2018.04.062.

[ref11] Weigel T.; Schipper F.; Erickson E. M.; Susai F. A.; Markovsky B.; Aurbach D. Structural and Electrochemical Aspects of LiNi 0.8 Co 0.1 Mn 0.1 O 2 Cathode Materials Doped by Various Cations. ACS Energy Lett. 2019, 4 (2), 508–516. 10.1021/acsenergylett.8b02302.

[ref12] Li T.; Yuan X. Z.; Zhang L.; Song D.; Shi K.; Bock C. Degradation Mechanisms and Mitigation Strategies of Nickel-Rich NMC-Based Lithium-Ion Batteries. Electrochemical Energy Reviews 2020, 3 (1), 43–80. 10.1007/s41918-019-00053-3.

[ref13] Deng S.; Wang B.; Yuan Y.; Li X.; Sun Q.; Doyle-Davis K.; Banis M. N.; Liang J.; Zhao Y.; Li J.; Li R.; Sham T. K.; Shahbazian-Yassar R.; Wang H.; Cai M.; Lu J.; Sun X. Manipulation of an Ionic and Electronic Conductive Interface for Highly-Stable High-Voltage Cathodes. Nano Energy 2019, 65, 103988 10.1016/j.nanoen.2019.103988.

[ref14] Zhang J.; Yang Z.; Gao R.; Gu L.; Hu Z.; Liu X. Suppressing the Structure Deterioration of Ni-Rich LiNi0.8Co0.1Mn0.1O2 through Atom-Scale Interfacial Integration of Self-Forming Hierarchical Spinel Layer with Ni Gradient Concentration. ACS Appl. Mater. Interfaces 2017, 9 (35), 29794–29803. 10.1021/acsami.7b08802. 28799736

[ref15] Wang H.; Chu Y.; Pan Q.; Yang G.; Lai A.; Liu Z.; Zheng F.; Hu S.; Huang Y.; Li Q. Enhanced Interfacial Reaction Interface Stability of Ni-Rich Cathode Materials by Fabricating Dual-Modified Layer Coating for Lithium-Ion Batteries. Electrochim. Acta 2021, 366, 137476 10.1016/j.electacta.2020.137476.

[ref16] Wang K. X.; Li X. H.; Chen J. S. Surface and Interface Engineering of Electrode Materials for Lithium-Ion Batteries. Adv. Mater. 2015, 27 (3), 527–545. 10.1002/adma.201402962. 25355133

[ref17] Kalluri S.; Yoon M.; Jo M.; Liu H. K.; Dou S. X.; Cho J.; Guo Z. Feasibility of Cathode Surface Coating Technology for High-Energy Lithium-Ion and Beyond-Lithium-Ion Batteries. Adv. Mater. 2017, 29 (48), 1605807 10.1002/adma.201605807. 28251710

[ref18] Lin F.; Markus I. M.; Nordlund D.; Weng T. C.; Asta M. D.; Xin H. L.; Doeff M. M. Surface Reconstruction and Chemical Evolution of Stoichiometric Layered Cathode Materials for Lithium-Ion Batteries. Nat. Commun. 2014, 5, 3529 10.1038/ncomms4529. 24670975

[ref19] Xu G. L.; Liu Q.; Lau K. K. S.; Liu Y.; Liu X.; Gao H.; Zhou X.; Zhuang M.; Ren Y.; Li J.; Shao M.; Ouyang M.; Pan F.; Chen Z.; Amine K.; Chen G. Building Ultraconformal Protective Layers on Both Secondary and Primary Particles of Layered Lithium Transition Metal Oxide Cathodes. Nat. Energy 2019, 4 (6), 484–494. 10.1038/s41560-019-0387-1.

[ref20] Cheng X.; Zheng J.; Lu J.; Li Y.; Yan P.; Zhang Y. Realizing Superior Cycling Stability of Ni-Rich Layered Cathode by Combination of Grain Boundary Engineering and Surface Coating. Nano Energy 2019, 62, 30–37. 10.1016/j.nanoen.2019.05.021.

[ref21] Meng X.; Yang X. Q.; Sun X. Emerging Applications of Atomic Layer Deposition for Lithium-Ion Battery Studies. *Advanced Materials*. Wiley-VCH Verlag 2012, 24 (27), 3589–3615. 10.1002/adma.201200397. 22700328

[ref22] Assaud L.; Pitzschel K.; Hanbücken M.; Santinacci L. Highly-Conformal TiN Thin Films Grown by Thermal and Plasma-Enhanced Atomic Layer Deposition. ECS Journal of Solid State Science and Technology 2014, 3 (7), P253–P258. 10.1149/2.0141407jss.

[ref23] Yang H.; Wu H. H.; Ge M.; Li L.; Yuan Y.; Yao Q.; Chen J.; Xia L.; Zheng J.; Chen Z.; Duan J.; Kisslinger K.; Zeng X. C.; Lee W. K.; Zhang Q.; Lu J. Simultaneously Dual Modification of Ni-Rich Layered Oxide Cathode for High-Energy Lithium-Ion Batteries. Adv. Funct Mater. 2019, 29 (13), 1808825 10.1002/adfm.201808825.

[ref24] Kim H.; Kim M. G.; Jeong H. Y.; Nam H.; Cho J. A New Coating Method for Alleviating Surface Degradation of LiNi0.6Co0.2Mn0.2O2 Cathode Material: Nanoscale Surface Treatment of Primary Particles. Nano Lett. 2015, 15 (3), 2111–2119. 10.1021/acs.nanolett.5b00045. 25668708

[ref25] Liu Y.; Liu W.; Zhu M.; Li Y.; Li W.; Zheng F.; Shen L.; Dang M.; Zhang J. Coating Ultra-Thin TiN Layer onto LiNi0.8Co0.1Mn0.1O2 Cathode Material by Atomic Layer Deposition for High-Performance Lithium-Ion Batteries. J. Alloys Compd. 2021, 888, 161594 10.1016/j.jallcom.2021.161594.

[ref26] Sun H. H.; Kim U. H.; Park J. H.; Park S. W.; Seo D. H.; Heller A.; Mullins C. B.; Yoon C. S.; Sun Y. K. Transition Metal-Doped Ni-Rich Layered Cathode Materials for Durable Li-Ion Batteries. Nat. Commun. 2021, 12 (1), 6552 10.1038/s41467-021-26815-6. 34772958 PMC8589951

[ref27] Tian C.; Lin F.; Doeff M. M. Electrochemical Characteristics of Layered Transition Metal Oxide Cathode Materials for Lithium Ion Batteries: Surface, Bulk Behavior, and Thermal Properties. Acc. Chem. Res. 2018, 51 (1), 89–96. 10.1021/acs.accounts.7b00520. 29257667

[ref28] Kim J.; Cho H.; Jeong H. Y.; Ma H.; Lee J.; Hwang J.; Park M.; Cho J. Self-Induced Concentration Gradient in Nickel-Rich Cathodes by Sacrificial Polymeric Bead Clusters for High-Energy Lithium-Ion Batteries. Adv. Energy Mater. 2017, 7 (12), 1602559 10.1002/aenm.201602559.

[ref29] Li S.; Fu X.; Zhou J.; Han Y.; Qi P.; Gao X.; Feng X.; Wang B. An Effective Approach to Improve the Electrochemical Performance of LiNi0.6Co0.2Mn0.2O2 Cathode by an MOF-Derived Coating. J. Mater. Chem. A Mater. 2016, 4 (16), 5823–5827. 10.1039/C5TA10773C.

[ref30] Sun D.; Sun F.; Deng X.; Li Z. Mixed-Metal Strategy on Metal-Organic Frameworks (MOFs) for Functionalities Expansion: Co Substitution Induces Aerobic Oxidation of Cyclohexene over Inactive Ni-MOF-74. Inorg. Chem. 2015, 54 (17), 8639–8643. 10.1021/acs.inorgchem.5b01278. 26288128

[ref31] Naaman R.; Waldeck D. H. Chiral-Induced Spin Selectivity Effect. J. Phys. Chem. Lett. 2012, 3 (16), 2178–2187. 10.1021/jz300793y. 26295768

[ref32] Naaman R.; Waldeck D. H. Spintronics and Chirality: Spin Selectivity in Electron Transport through Chiral Molecules. *Annual Review of Physical Chemistry*. Annual Reviews Inc. April 1 2015, 66, 263–281. 10.1146/annurev-physchem-040214-121554. 25622190

[ref33] Naaman R.; Paltiel Y.; Waldeck D. H. Chiral Molecules and the Electron Spin. Nat. Rev. Chem. 2019, 3, 250–260. 10.1038/s41570-019-0087-1.

[ref34] Evers F.; Aharony A.; Bar-Gill N.; Entin-Wohlman O.; Hedegård P.; Hod O.; Jelinek P.; Kamieniarz G.; Lemeshko M.; Michaeli K.; Mujica V.; Naaman R.; Paltiel Y.; Refaely-Abramson S.; Tal O.; Thijssen J.; Thoss M.; van Ruitenbeek J. M.; Venkataraman L.; Waldeck D. H.; Yan B.; Kronik L. Theory of Chirality Induced Spin Selectivity: Progress and Challenges. Adv. Mater. 2022, 34 (13), 2106629 10.1002/adma.202106629. 35064943

[ref35] Kumar A.; Capua E.; Kesharwani M. K.; Martin J. M. L.; Sitbon E.; Waldeck D. H.; Naaman R. Chirality-Induced Spin Polarization Places Symmetry Constraints on Biomolecular Interactions. Proc. Natl. Acad. Sci. U. S. A. 2017, 114 (10), 2474–2478. 10.1073/pnas.1611467114. 28228525 PMC5347616

[ref36] Michaeli K.; Kantor-Uriel N.; Naaman R.; Waldeck D. H. The Electron’s Spin and Molecular Chirality-How Are They Related and How Do They Affect Life Processes?. Chem. Soc. Rev. 2016, 45, 6478–6487. 10.1039/C6CS00369A. 27734046

[ref37] Metzger T. S.; Mishra S.; Bloom B. P.; Goren N.; Neubauer A.; Shmul G.; Wei J.; Yochelis S.; Tassinari F.; Fontanesi C.; Waldeck D. H.; Paltiel Y.; Naaman R. The Electron Spin as a Chiral Reagent. Angew. Chem. 2020, 132 (4), 1670–1675. 10.1002/ange.201911400. 31621990

[ref38] Ghosh S.; Mishra S.; Avigad E.; Bloom B. P.; Baczewski L. T.; Yochelis S.; Paltiel Y.; Naaman R.; Waldeck D. H. Effect of Chiral Molecules on the Electron’s Spin Wavefunction at Interfaces. J. Phys. Chem. Lett. 2020, 11 (4), 1550–1557. 10.1021/acs.jpclett.9b03487. 32013436 PMC7307953

[ref39] Michaeli K.; Naaman R. Origin of Spin-Dependent Tunneling Through Chiral Molecules. J. Phys. Chem. C 2019, 123 (27), 17043–17048. 10.1021/acs.jpcc.9b05020.

[ref40] Ziv A.; Saha A.; Alpern H.; Sukenik N.; Baczewski L. T.; Yochelis S.; Reches M.; Paltiel Y. AFM-Based Spin-Exchange Microscopy Using Chiral Molecules. Adv. Mater. 2019, 31 (40), 1904206 10.1002/adma.201904206. 31423697

[ref41] Roushan P.; Seo J.; Parker C. V.; Hor Y. S.; Hsieh D.; Qian D.; Richardella A.; Hasan M. Z.; Cava R. J.; Yazdani A. Topological Surface States Protected from Backscattering by Chiral Spin Texture. Nature 2009, 460 (7259), 1106–1109. 10.1038/nature08308. 19668187

[ref42] Mtangi W.; Tassinari F.; Vankayala K.; Vargas Jentzsch A.; Adelizzi B.; Palmans A. R. A.; Fontanesi C.; Meijer E. W.; Naaman R. Control of Electrons’ Spin Eliminates Hydrogen Peroxide Formation during Water Splitting. J. Am. Chem. Soc. 2017, 139 (7), 2794–2798. 10.1021/jacs.6b12971. 28132505 PMC5330654

[ref43] Kapon Y.; Saha A.; Duanis-Assaf T.; Stuyver T.; Ziv A.; Metzger T.; Yochelis S.; Shaik S.; Naaman R.; Reches M.; Paltiel Y. Evidence for New Enantiospecific Interaction Force in Chiral Biomolecules. Chem. 2021, 7 (10), 2787–2799. 10.1016/j.chempr.2021.08.002.

[ref44] Mtangi W.; Kiran V.; Fontanesi C.; Naaman R. Role of the Electron Spin Polarization in Water Splitting. J. Phys. Chem. Lett. 2015, 6 (24), 4916–4922. 10.1021/acs.jpclett.5b02419. 26615833 PMC4685426

[ref45] Zhang W.; Banerjee-Ghosh K.; Tassinari F.; Naaman R. Enhanced Electrochemical Water Splitting with Chiral Molecule-Coated Fe3O4 Nanoparticles. ACS Energy Lett. 2018, 3 (10), 2308–2313. 10.1021/acsenergylett.8b01454.

[ref46] Ahn J.; Yim T. Ni-Rich LiNi0.8Co0.1Mn0.1O2 Oxide Functionalized by Allyl Phenyl Sulfone as High-Performance Cathode Material for Lithium-Ion Batteries. J. Alloys Compd. 2021, 867, 159153 10.1016/j.jallcom.2021.159153.

[ref47] Amsallem D.; Kumar A.; Naaman R.; Gidron O. Spin Polarization through Axially Chiral Linkers: Length Dependence and Correlation with the Dissymmetry Factor. Chirality 2023, 35, 562–568. 10.1002/chir.23556. 36896481

[ref48] Ghosh S.; Bloom B. P.; Lu Y.; Lamont D.; Waldeck D. H. Increasing the Efficiency of Water Splitting through Spin Polarization Using Cobalt Oxide Thin Film Catalysts. J. Phys. Chem. C 2020, 124 (41), 22610–22618. 10.1021/acs.jpcc.0c07372.

[ref49] Naaman R.; Paltiel Y.; Waldeck D. H. Chiral Induced Spin Selectivity Gives a New Twist on Spin-Control in Chemistry. Acc. Chem. Res. 2020, 53 (11), 2659–2667. 10.1021/acs.accounts.0c00485. 33044813 PMC7676290

[ref50] Naaman R.; Paltiel Y.; Waldeck D. H. Chiral Molecules and the Electron Spin. Nature Reviews Chemistry 2019, 3 (4), 250–260. 10.1038/s41570-019-0087-1.

