# Supplementary material for: Chiral Molecular Coating of a LiNiCoMnO2 Cathode for High-Rate Capability Lithium-Ion Batteries
Source: J Phys Chem Lett. 2024 Mar 1;15(10):2682–9. doi: 10.1021/acs.jpclett.4c00171 (PMC10945569; doi:10.1021/acs.jpclett.4c00171)
Supplement: Supplementary file 1 — jz4c00171_si_001.pdf [file jz4c00171_si_001.pdf]

Chiral Molecular Coating of a $LiNiCoMnO_2$ Cathode for High-Rate Capability Lithium-Ion Batteries

Nir Yuran#, Bagavathi Muniyandi#, Arka Saha#, Shira Yochelis, Daniel Sharon*, Yossi Paltiel*, Malachi Noked*

Department of Applied Physics, Center for Nanoscience and Nanotechnology, Hebrew University of Jerusalem, Jerusalem, 91904, Israel

Department of Chemistry, Bar Ilan Institute for Nanotechnology and Advanced Materials, Bar Ilan University, Ramat Gan, 5290002, Israel

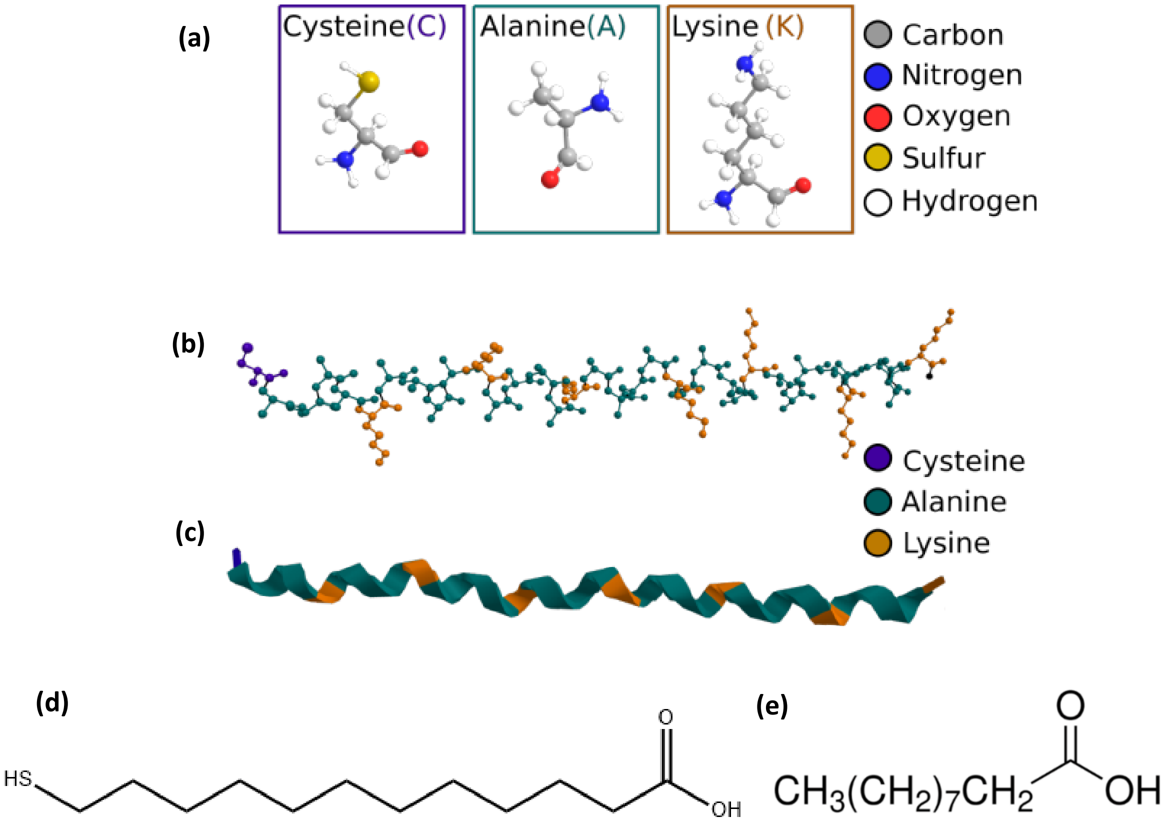

Fig. S1- Structure of the peptides used in this study. (a) the building blocks Cysteine (C), Alanine (A) and Lysine (K) composing the peptide AHPA36. (b) Atomic structure of 36 monomers polypeptide with C[AAAAK]7. (c) Schematic view of the helical secondary structure. A 36-mer comprises 10 windings with a pitch size of 5.4 A, i.e. 3.6 A unit. The different peptide units are labeled by different colors. (d, e) 12-mercaptododecanic acid structure and chemical formula, respectively.

Taken from reference 1 with permission American Physical Society.

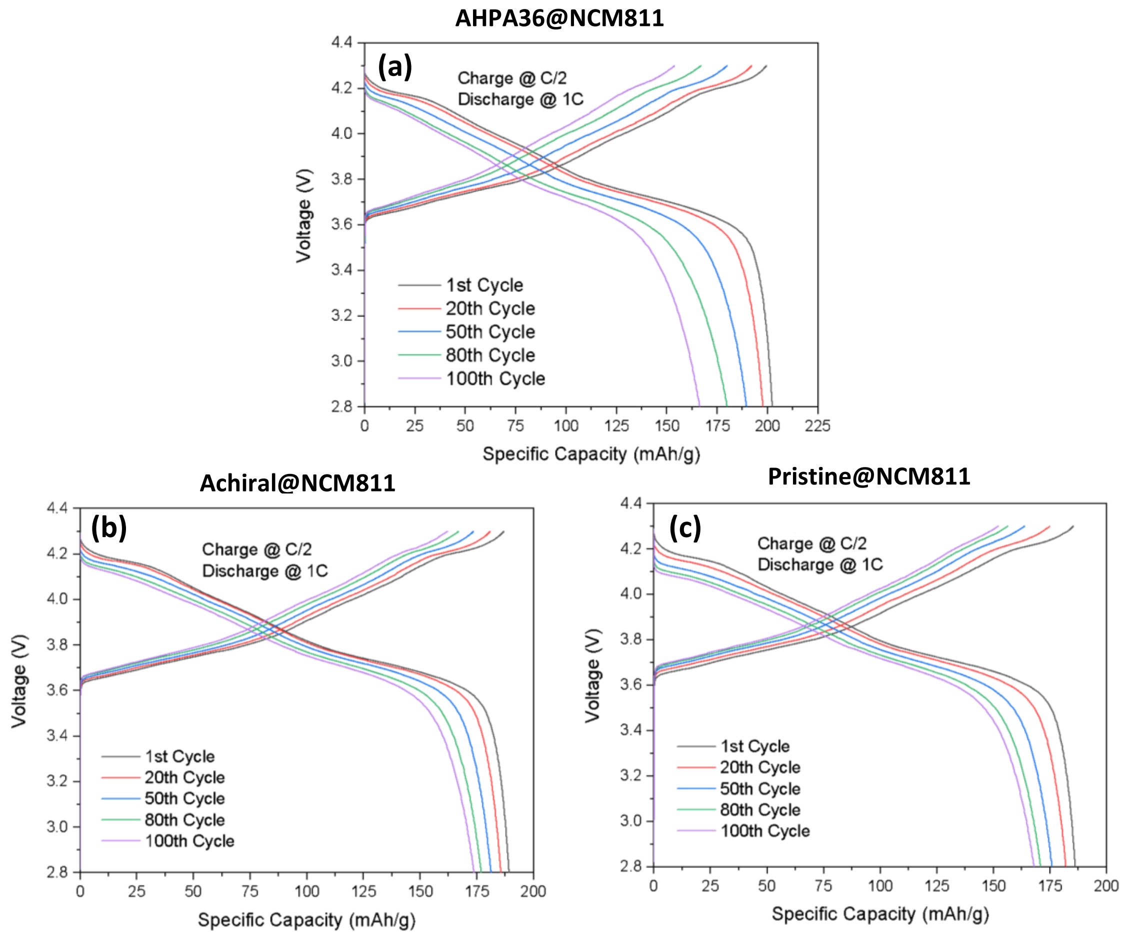

Fig. S2 Typical voltage-specific capacity profiles of the (a) AHPA36@NCM811, (b) Achiral@NCM811, (c) Pristine NCM811 cathodes during prolong cycles at 1C rate in half-cell (vs. Li/Li^+) at 30 ^{0}C.

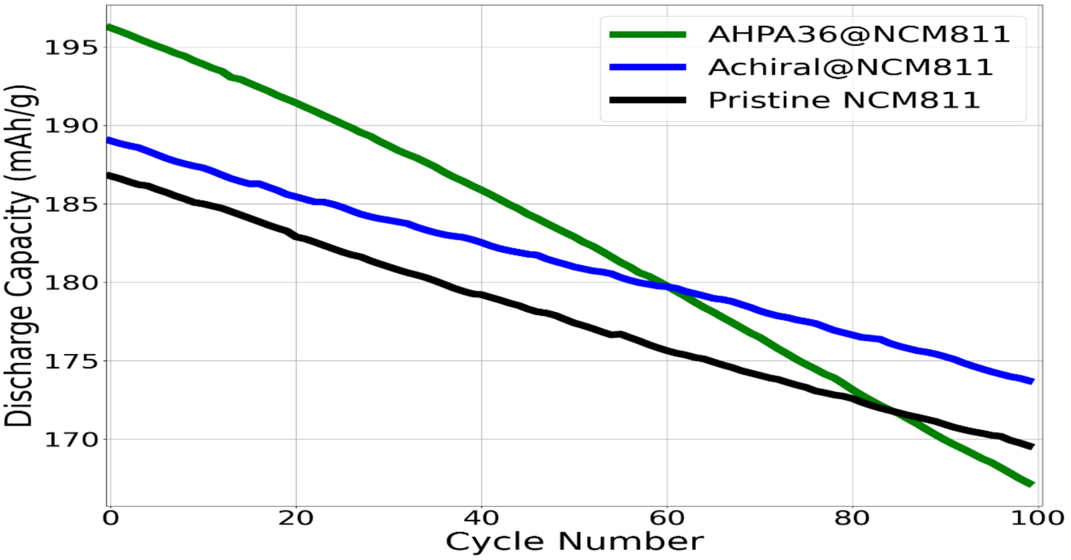

Fig. S3 Cyclic stability performance 1C-rate. All the above measurements were performed at 35 °C in 1 M LiPF6 EC/EMC (3:7) electrolyte solution.

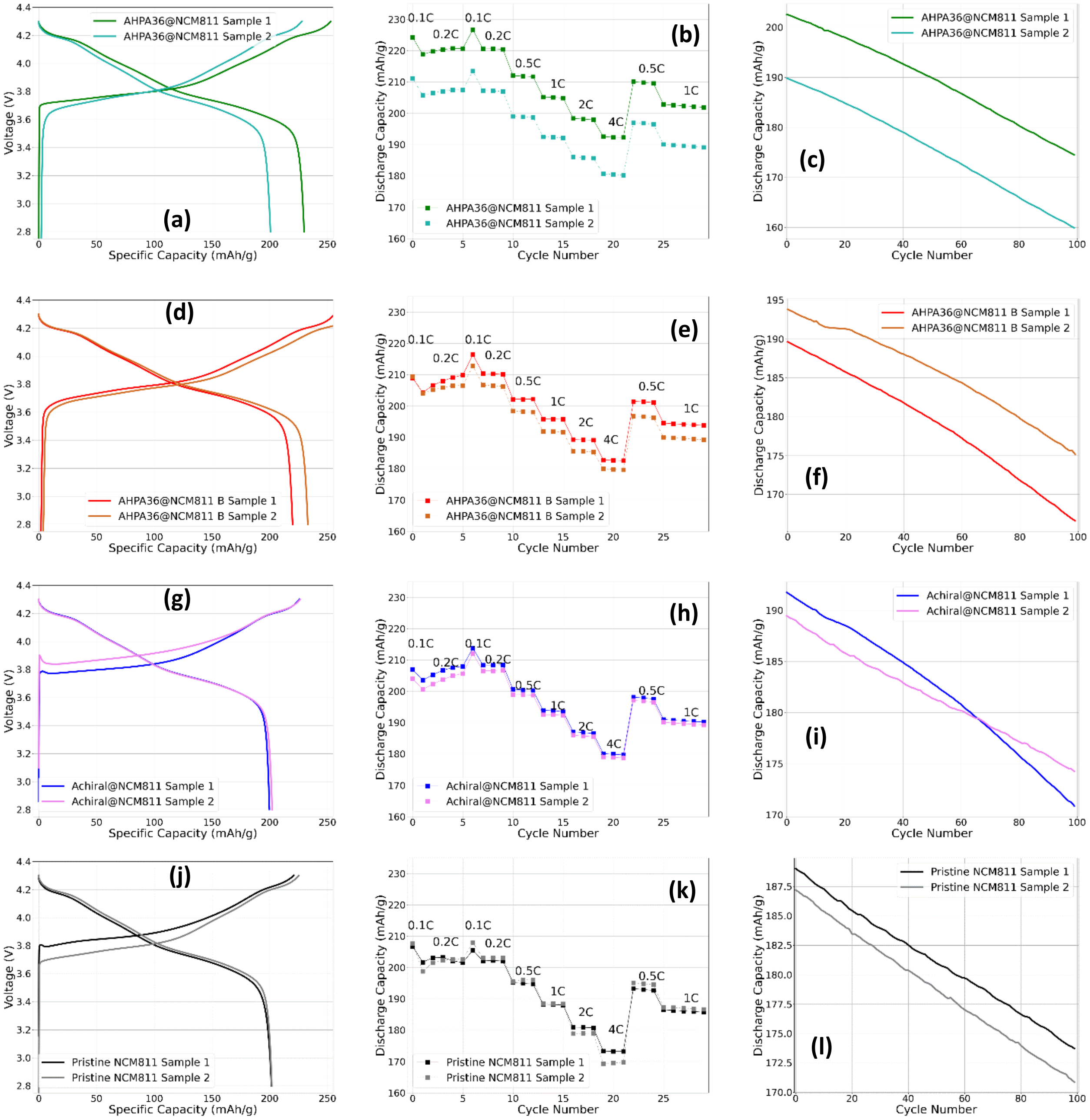

Fig. S4: Voltage profile, Rate study, and long cycling performance, of AHPA36@NCM811 (a, b, c), AHPA36@NCM811 B (d, e, f), Achiral@NCM811 (g, h, i), and Pristine NCM811 (j, k, l), respectively.

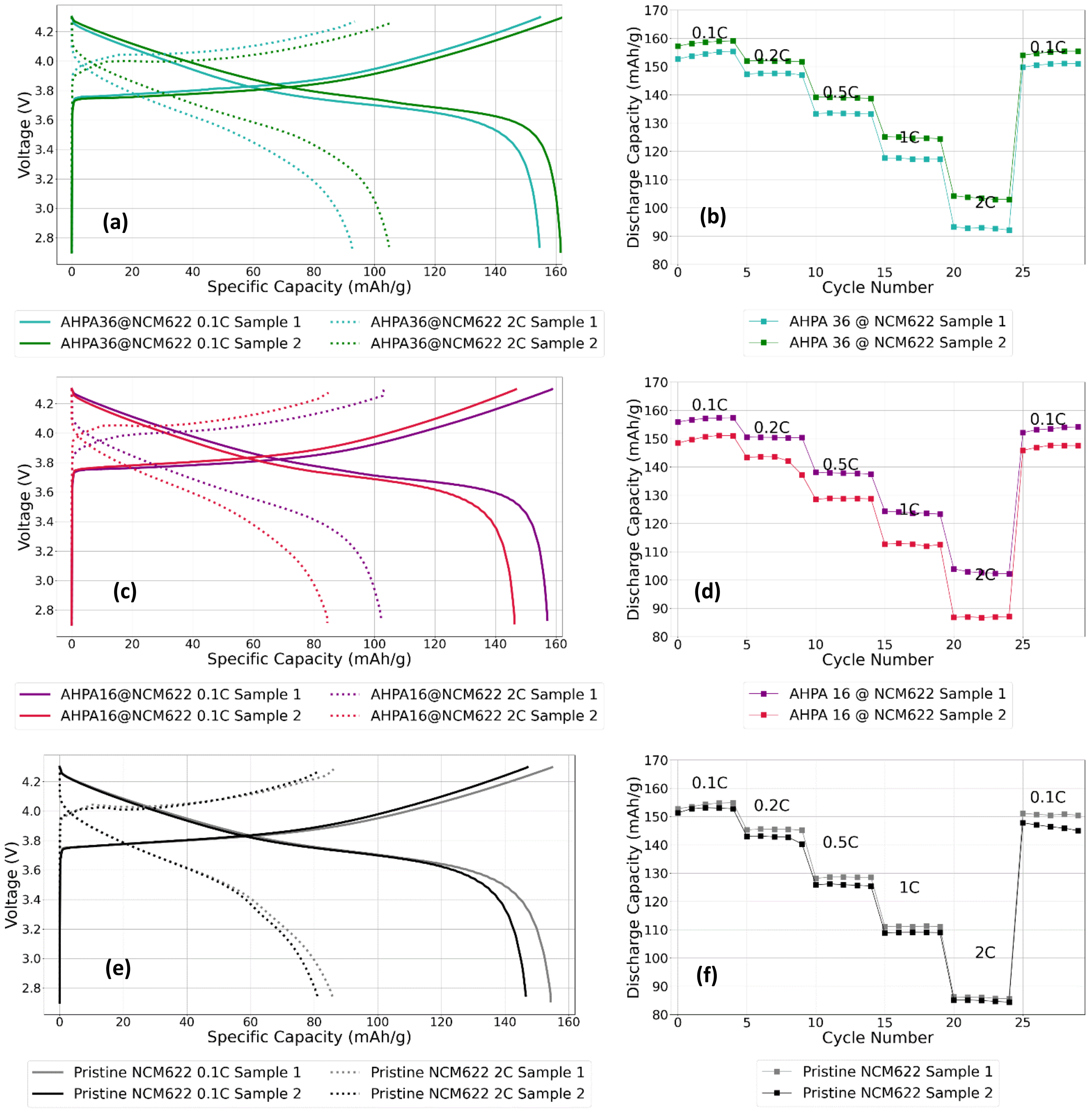

Fig. S5: Voltage profile and Rate Study of AHPA36@NCM622 (a, b), AHPA16@NCM622 (c, d), and Pristine NCM622 (e, f), respectively.

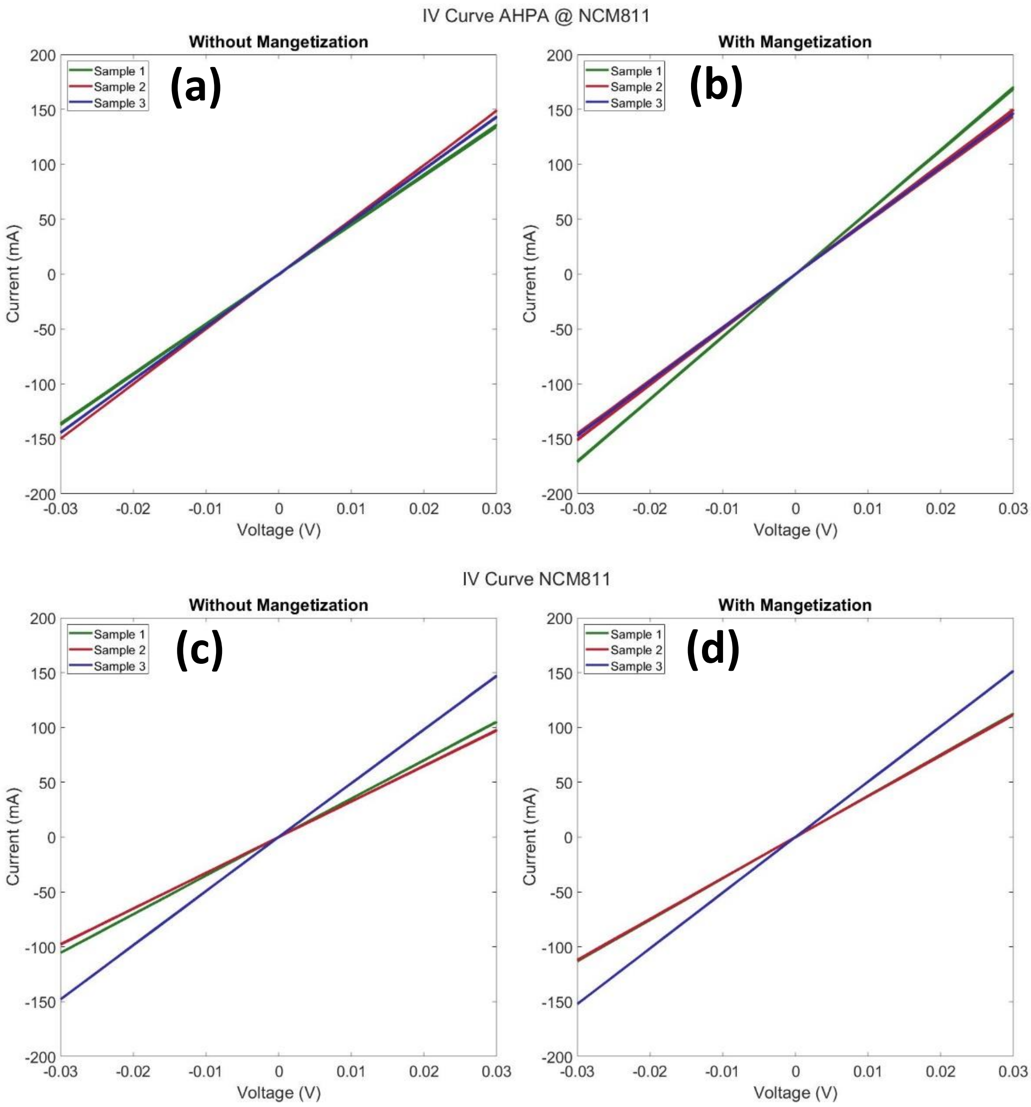

Fig. S6: IV curve of AHPA36 @ NCM811 and Pristine NCM811 with Ni electrode on top and beneath, with and without magnetization.

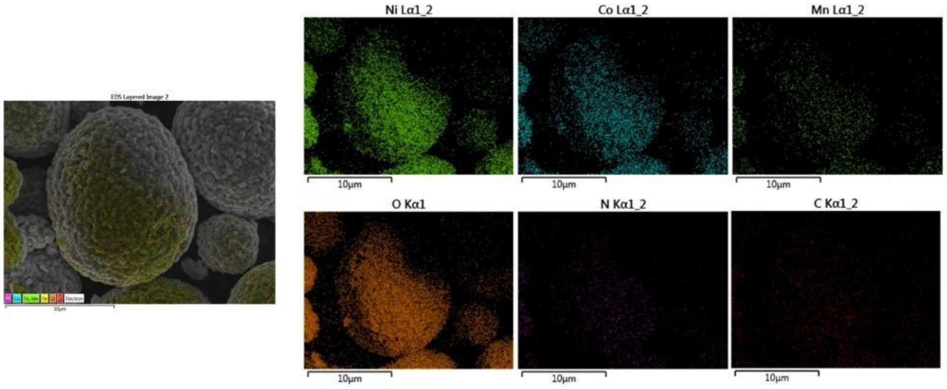

Fig. S7 EDS mapping images of AHPA36@NCM811 with coloring the elements Ni, Co, Mn, O, N and C.

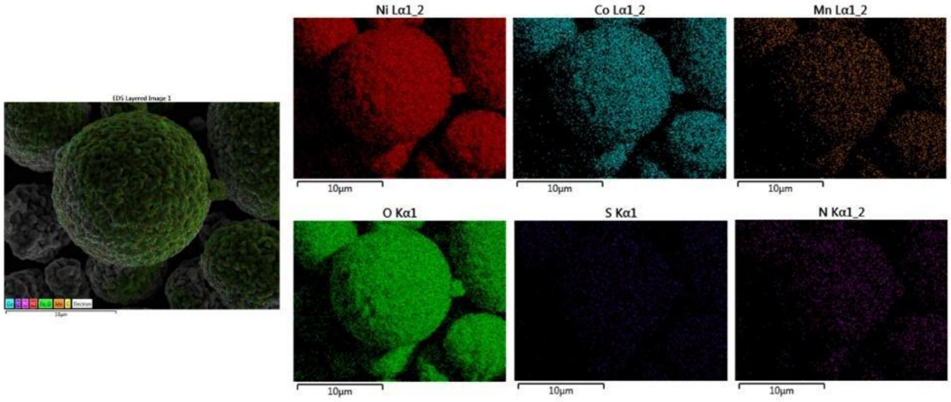

Fig. S8 EDS mapping images of Achiral@NCM811 with coloring the elements Ni, Co, Mn, O, N, S and C.

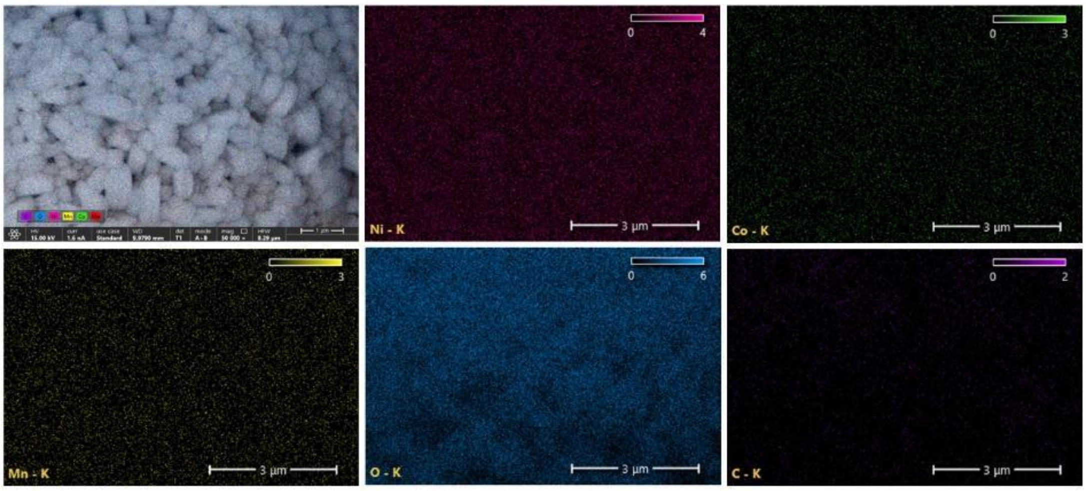

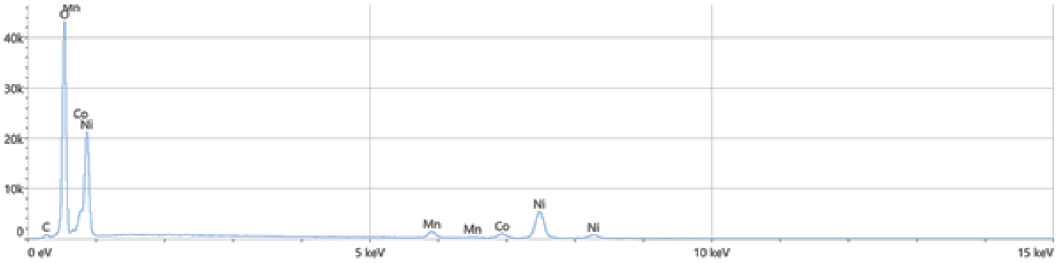

Element	Weight %	Atomic %
C	0.4	1.0
O	35.8	66.5
Mn	5.2	2.8
Co	6.8	3.4
Ni	51.6	26.2

Fig. S9 EDS mapping images of Pristine-NCM811 with coloring the elements Ni, Co, Mn, O and C.

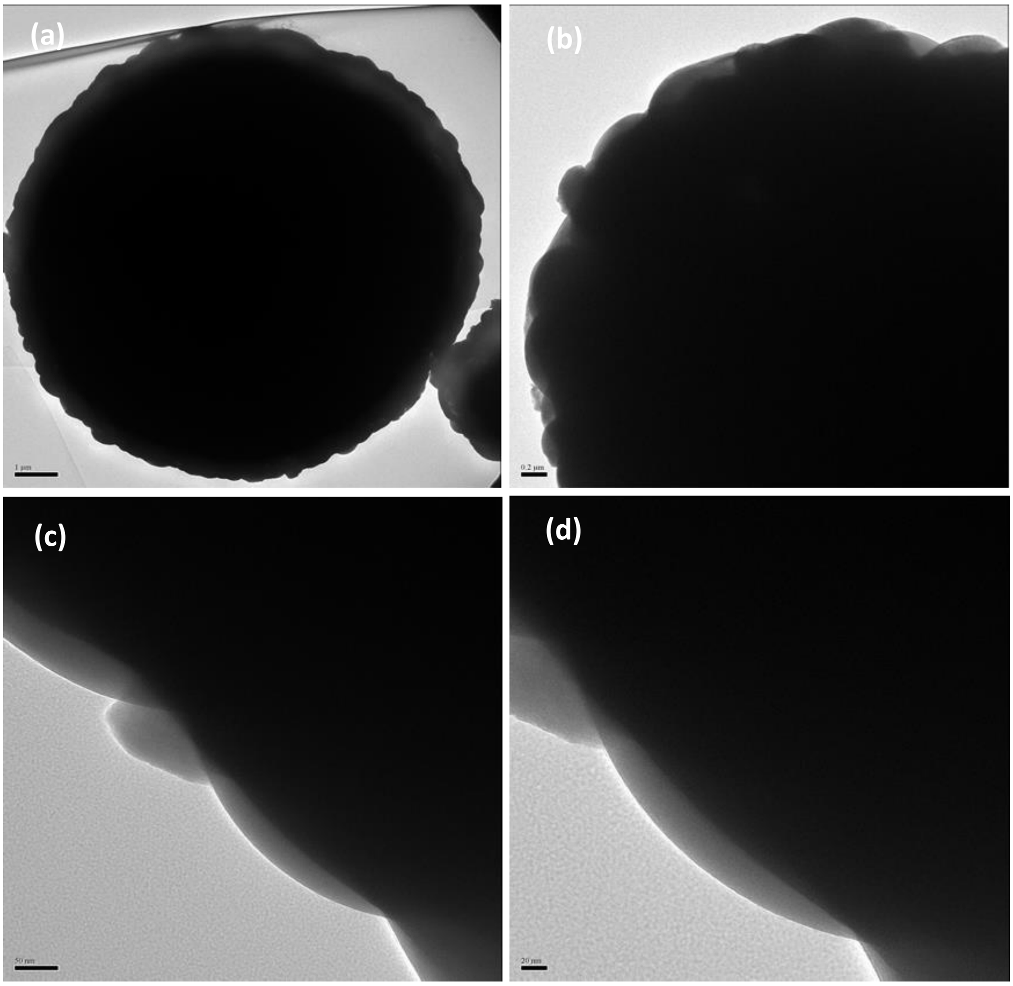

Fig. S10 (a-d) Low and high magnification TEM images of AHPA36@NMC811

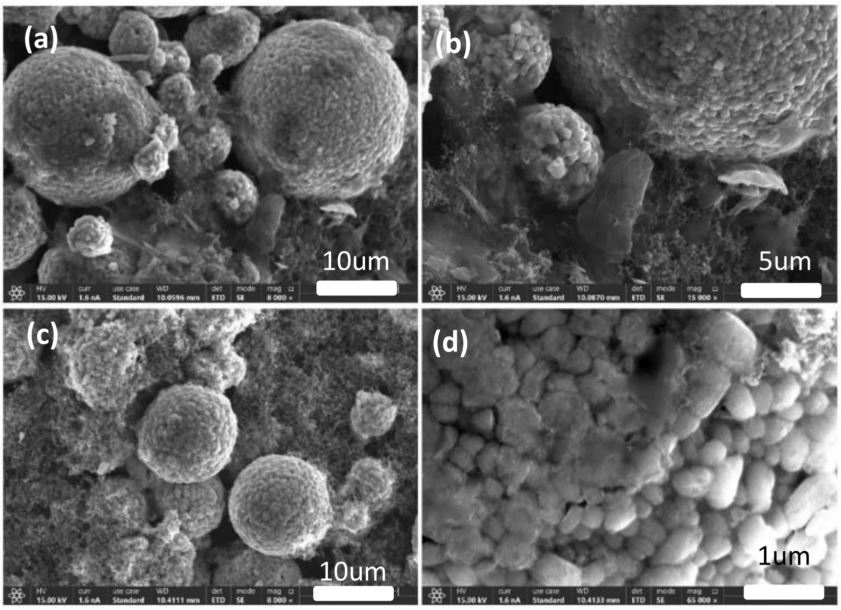

Fig. S11 Low and high magnification HRSEM images of Chiral A/NCM a & b - pre-cycling and c&d forpost-cycling electrodes.

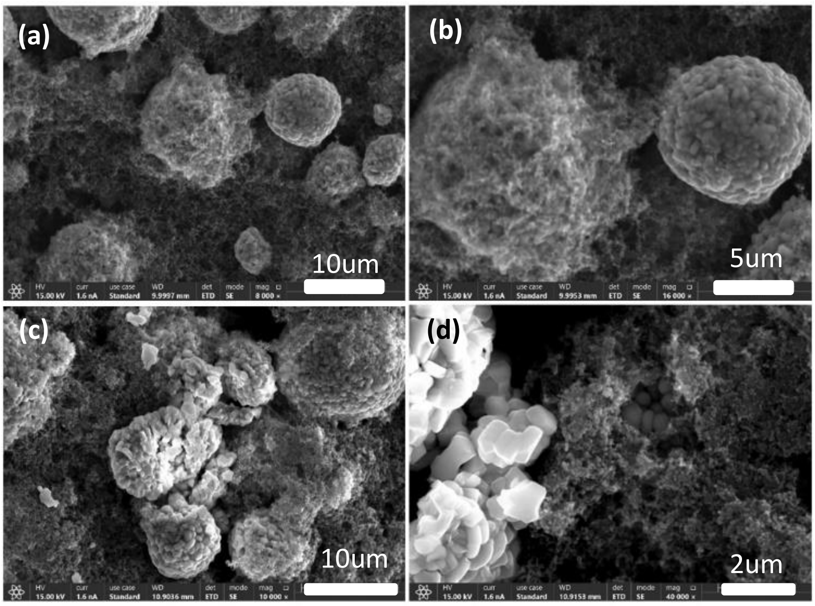

Fig. S12 Low and high magnification HRSEM images of pristine-NCM811 (a & b) pre-cycling, and (c&d) post-cycling electrodes.

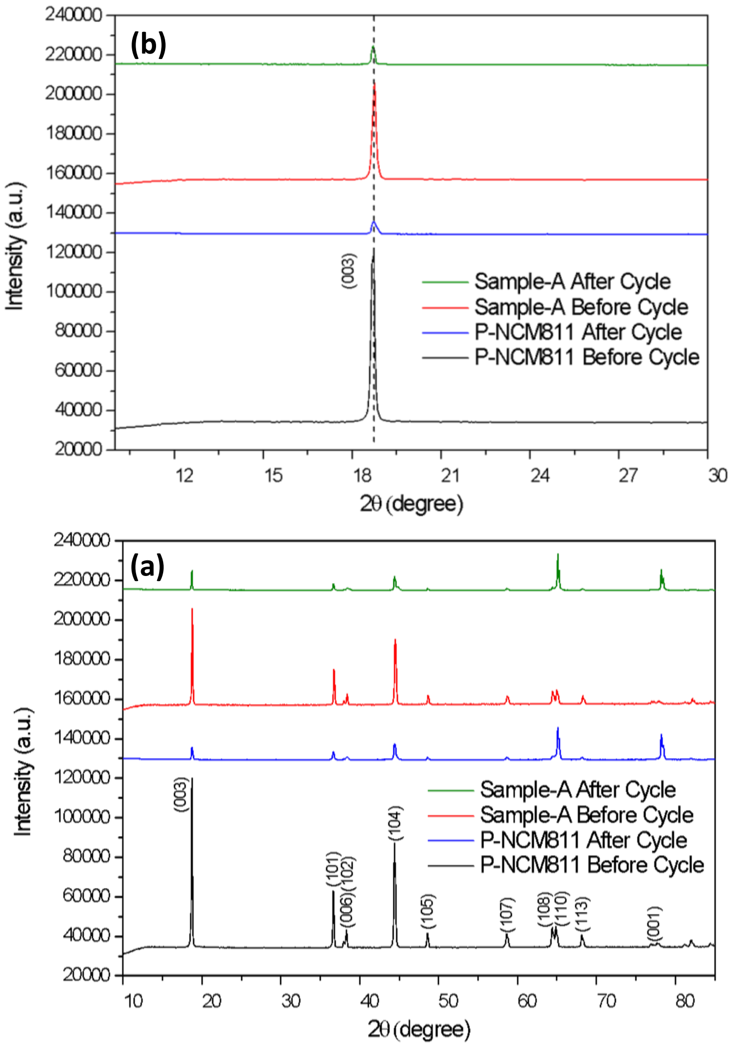

Fig. S13 Comparative XRD patterns of the pre and post-cycling cathodes of Treated Chiral-A/NCM and P-NCM811.

(1) Diana Slawig, Thi Ngoc Ha Nguyen, Shira Yochelis, Yossi Paltiel, and Christoph Tegenkamp. Electronic Transport Through Single Polyalanine Molecules. Phys. Rev. B, **2020**, 102(11), 115425
